# Stage-specific signaling modulation generates low-tumorigenicity thyroid progenitors from human pluripotent stem cells

**DOI:** 10.3389/fendo.2026.1885613

**Published:** 2026-08-31

**Authors:** Lili Dong, Manzhen Peng, Chuanyu Yao, Xie Cheng, Haisong Liu

**Affiliations:** 1 School of Biomedical Sciences, Hunan University, Changsha, Hunan, China; 2 Xiangtan Central Hospital, Affiliated to Hunan University, Xiangtan, Hunan, China

**Keywords:** direct differentiation, FOXE1, hPSCs, thyroid progenitors, thyroid regeneration

## Abstract

**Introduction:**

Human pluripotent stem cell (hPSC)-derived thyroid cells hold great promise for thyroid disease modeling, drug screening, and cell replacement therapies. However, the direct differentiation of hPSCs into functional thyroid follicles remains challenging, suggesting deficient specification of hPSC-derived progenitor cells in conventional protocols.

**Methods:**

In this study, we utilized a *PAX8*-mCherry hPSC reporter line to evaluate early differentiation and applied systematic pathway screening to optimize induction conditions. Furthermore, early-stage hPSC induction conditions were refined, and in vivo transplantation was performed to assess morphogenic capacity.

**Results:**

We observed that inadequate *FOXE1* induction may contribute to the limited maturation capacity of conventional protocols. Through our systematic screening, we successfully identified the optimized conditions required for generating NKX2-1/PAX8/FOXE1 triple-positive thyroid progenitors from hPSCs. Notably, these hPSC-derived progenitors are capable of forming thyroglobulin-positive follicular structures upon *in vivo* transplantation. Furthermore, we significantly reduced the inherent tumorigenic risk associated with residual hPSCs.

**Discussion:**

In summary, we present an efficient approach to direct hPSCs into low-tumorigenicity thyroid progenitors capable of engraftment and early *in vivo* morphogenesis, establishing a robust platform for thyroid regenerative medicine.

## Introduction

Thyroid disorders pose a profound global health burden, affecting an estimated 200 million people worldwide (1, 2). These disorders encompass a wide spectrum of conditions, including hypothyroidism, autoimmune thyroid diseases, thyroid nodules, and thyroid cancer (3, 4). Epidemiological studies have established strong associations between thyroid dysfunction and various systemic conditions, such as diabetes and cardiovascular disease, leading to increased morbidity and mortality (5–10). While levothyroxine replacement remains the standard of care for hypothyroidism, a significant proportion of patients do not respond optimally and face long-term health risks (11, 12). A fundamental impediment to advancing thyroid research is the lack of robust *in vitro* human models, which hampers the elucidation of disease mechanisms and the development of targeted therapeutics. Consequently, there is an urgent need for innovative strategies to generate functional thyroid follicular cells (TFCs) for disease modeling, drug discovery, and regenerative medicine.

Human pluripotent stem cells (hPSCs) offer a transformative platform to bypass the limitations inherent to primary TFC isolation, such as donor scarcity and high batch-to-batch variability. Recently, significant strides have been made in the development of 3D thyroid organoid technologies, which better recapitulate the spatial architecture and apico-basal polarization essential for follicular assembly and functional maturation (13–15). However, although stepwise differentiation protocols have successfully generated thyroid progenitors (TPs) co-expressing NKX2–1 and PAX8, these hPSC-derived cells typically fail to achieve terminal functional maturation—particularly the capacity for *de novo* thyroid hormone synthesis—following transplantation (16). Beyond functional immaturity, the clinical translation of hPSC-derived grafts is severely impeded by oncogenic risks. The persistence of even trace amounts of residual undifferentiated cells poses a critical risk of *in vivo* teratoma formation (17). Therefore, a viable regenerative strategy must concurrently guarantee robust hormonal functionality and the substantial reduction of tumorigenic risk—a dual objective that has remained a long-standing challenge in the field.

The embryonic specification of the thyroid gland relies on the precise co-expression of core thyroid transcription factors, including *NKX2-1*, *PAX8*, and *FOXE1*, which collectively dictate progenitor commitment and functional differentiation (18). This developmental trajectory is governed by a tightly orchestrated network of signaling pathways—including Wnt, BMP, FGF, and Hedgehog (Hh)—which coordinate the precise patterning of the anterior foregut endoderm (AFE) prior to definitive thyroid lineage commitment (19–21). Among the core thyroid transcription factors, *Foxe1* is uniquely indispensable for morphogenesis and follicular organization, as demonstrated in murine models. During mouse embryogenesis, *Foxe1* is required for the migration of the thyroid anlage, and its *in vivo* knockout (*Foxe1*^−/−^) results in severe thyroid dysgenesis (22). Furthermore, recent *in vitro* studies using mouse embryonic stem cell-derived organoids have confirmed that *Foxe1* deficiency impairs thyroid fate determination (23). Additionally, its disruption severely impairs the proper transcriptional maintenance of thyroid-specific functional genes, such as *Tg* and *Tpo (*22, 24, 25). However, despite its established *in vivo* role in mammals, the specific function and temporal requirements of *FOXE1* in human pluripotent stem cell (hPSC) differentiation remain poorly understood. While current protocols successfully induce *NKX2–1* and *PAX8*, they consistently fail to robustly activate or sustain *FOXE1* expression (20, 26). This critical gap in recapitulating the full thyroid transcription factor network remains a major obstacle to achieving fully mature, hPSC-derived thyroid lineages.

Building on our expertise in stage-specific chemical modulation (27), we developed a robust protocol to generate hPSC-derived TPs with improved *in vivo* safety. Optimizing the AFE window synchronized the induction of NKX2-1, PAX8, and FOXE1 and substantially improved the *in vivo* safety profile. Upon transplantation, these progenitors organized into thyroglobulin-positive follicular structures. Definitive functional maturation remains to be demonstrated in a hypothyroid recipient model.

## Results

### Conventional protocols establish early thyroid progenitor identity but limit terminal differentiation

**Figure 1:**
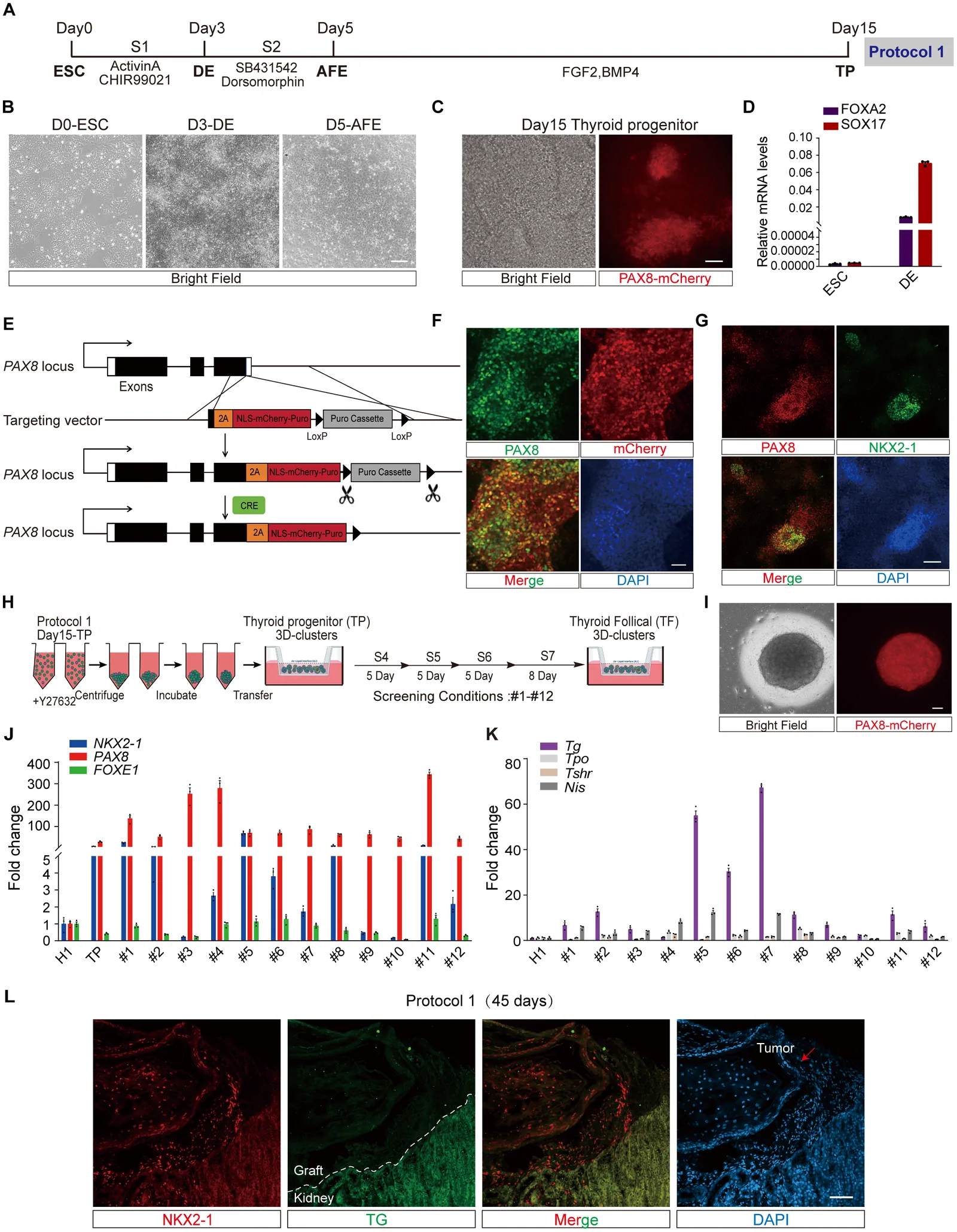
Directed differentiation of thyroid progenitors via Protocol 1 reveals *in vitro* maturation potential and *in vivo* limitations. **(A)** Schematic of the standard three-stage thyroid progenitor differentiation (Protocol 1). DE, definitive endoderm; AFE, anterior foregut endoderm; TP, thyroid progenitor. **(B)** Representative bright-field images depicting the stepwise morphological progression from human embryonic stem cells (ESC) through definitive endoderm (DE) to anterior foregut endoderm (AFE) using Protocol 1. Scale bar, 300 μm. **(C)** Day 15 thyroid progenitors generated under Protocol 1; left, bright-field; right, endogenous *PAX8*-mCherry fluorescence demonstrating efficient reporter activation. Scale bar, 100 μm. **(D)** RT-qPCR analysis of definitive endoderm markers (*FOXA2* and *SOX17*) in H1 and DE cells. Data are presented as mean ± SD (n = 3). **(E)** Targeting strategy for the CRISPR/Cas9-engineered *PAX8*-mCherry knock-in reporter line. **(F)** Representative immunofluorescence showing co-localization of *PAX8*-mCherry fluorescence with endogenous PAX8 protein on Day 15 of differentiation. Scale bars, 50 μm. **(G)** Immunofluorescence of Day 15 cells from Protocol 1, showing co-expression of NKX2–1 and PAX8. Nuclei are labeled with DAPI. Scale bars, 100 μm. **(H)** Schematic of the air-liquid interface (ALI) culture system employed to promote *in vitro* maturation of Day 15 thyroid progenitors. **(I)** *PAX8*-mCherry^+^ clusters observed after ALI culture, indicative of progenitor cell aggregation. Scale bar, 30 μm. **(J)** RT-qPCR analysis of the indicated thyroid lineage markers (*NKX2-1*, *PAX8*, *FOXE1*) in Day 15 TPs and after maturation under conditions #1– #12 using Protocol 1. Data represent mean ± SD (n = 3). **(K)** RT-qPCR analysis of the indicated thyroid maturation markers in cells subjected to maturation conditions #1– #12 for 23 days. Transcript levels are shown as fold change relative to undifferentiated H1 ESCs. Data represent mean ± SD (n = 3). **(L)** Representative immunofluorescence images of kidney grafts at Day 45 post-transplantation of Day 15 progenitors derived from Protocol 1. Red arrows indicate tumor regions. Tumors were defined as described in the main text. Dashed lines delineate the boundary between the host kidney and the graft. Scale bars, 100 μm.

To dynamically monitor thyroid lineage commitment from hPSCs, we generated a CRISPR/Cas9-mediated *PAX8*-mCherry knock-in reporter line (**Figure 1E**), which precisely recapitulates PAX8 expression during differentiation (**Figure 1F**). Utilizing a conventional three-stage differentiation paradigm (designated as Protocol 1; **Figure 1A**), we observed a stepwise morphological transition from definitive endoderm (DE) to anterior foregut endoderm (AFE) (**Figure 1B**). Stage-wise identity was confirmed by RT-qPCR: the pluripotency of the *PAX8*-mCherry reporter subclone was confirmed by expression of the core pluripotency factors *OCT4* and *NANOG* (**Supplementary Figure 1A**), definitive endoderm markers *FOXA2* and *SOX17* were induced in DE cells relative to undifferentiated *PAX8*-mCherry cells (**Figure 1D**), and anterior foregut endoderm markers were correspondingly upregulated at the AFE stage (**Supplementary Figure 1B**), successfully yielding *PAX8*-mCherry^+^ progenitors by Day 15 (**Figure 1C**). Although these Day 15 cells exhibited robust co-expression of the core thyroid transcription factors NKX2–1 and PAX8 (**Figure 1G**), the expression of FOXE1—a critical regulator of thyroid morphogenesis—remained negligible, with transcript levels comparable to those of undifferentiated hESCs (**Figure 1J**). Notably, this FOXE1 deficiency was refractory to a broad panel of screening conditions applied during the subsequent *in vitro* maturation phase (#1–#12; **Supplementary Figure 1C**; **Figure 1J**), suggesting that Protocol 1-derived progenitors fail to activate the crucial gene regulatory network required for terminal maturation.

To investigate the functional maturation potential of Protocol 1-derived progenitors *in vitro*, we subjected these cells to air–liquid interface (ALI) culture (**Figure 1H**). Despite the formation of three-dimensional (3D) mCherry^+^ clusters (**Figure 1I**), quantitative transcriptomic analysis revealed that, apart from a marginal upregulation of thyroglobulin (*TG*) transcripts, most terminal thyroid differentiation markers remained quiescent (**Figure 1K**). To evaluate their long-term developmental potential *in vivo*, Day 15 progenitors were transplanted beneath the kidney capsule of immunodeficient mice. For all transplantation experiments, tumors were defined as macroscopic nodules (>1 cm in diameter) visible at graft harvest, with their teratomatous nature subsequently confirmed by histological examination showing irregular cellular proliferation and disorganized tissue architecture comprising derivatives of all three germ layers. Consistent with our *in vitro* findings, the grafts failed to organize into TG-positive follicular structures by Day 45 (**Figure 1L**), and no organized TG^+^ follicular structures were observed even at Day 90 post-transplantation (**Supplementary Figure 1D**). Collectively, these data demonstrate that while the conventional Protocol 1 successfully initiates early thyroid fate specification, it is insufficient to drive the comprehensive transcriptional program required for functional follicular morphogenesis.

### Stage-specific DAPT and RepSox treatments partially enhance thyroid progenitor specification

Although *Foxe1* acts as a Shh/Gli-responsive gene in certain epithelial tissues—such as the hair follicle, where Gli2 indirectly activates the *Foxe1* promoter (28)—its induction in the thyroid is largely independent of direct Hedgehog signaling. Indeed, Shh-null mice maintain thyroidal expression of *Nkx2-1, Foxe1*, and thyroglobulin (Tg), indicating that Shh primarily influences thyroid morphogenesis through indirect mechanisms (21, 29). To determine whether pharmacological Hedgehog activation could nonetheless enhance FOXE1 induction in our *in vitro* human system, we supplemented the cultures with the Hedgehog agonist SAG during Stage 3 (S3). However, this treatment failed to substantially increase the proportion of *PAX8*-mCherry^+^ cells or upregulate core thyroid transcription factors (**Supplementary Figures 2A, B**).

**Figure 2:**
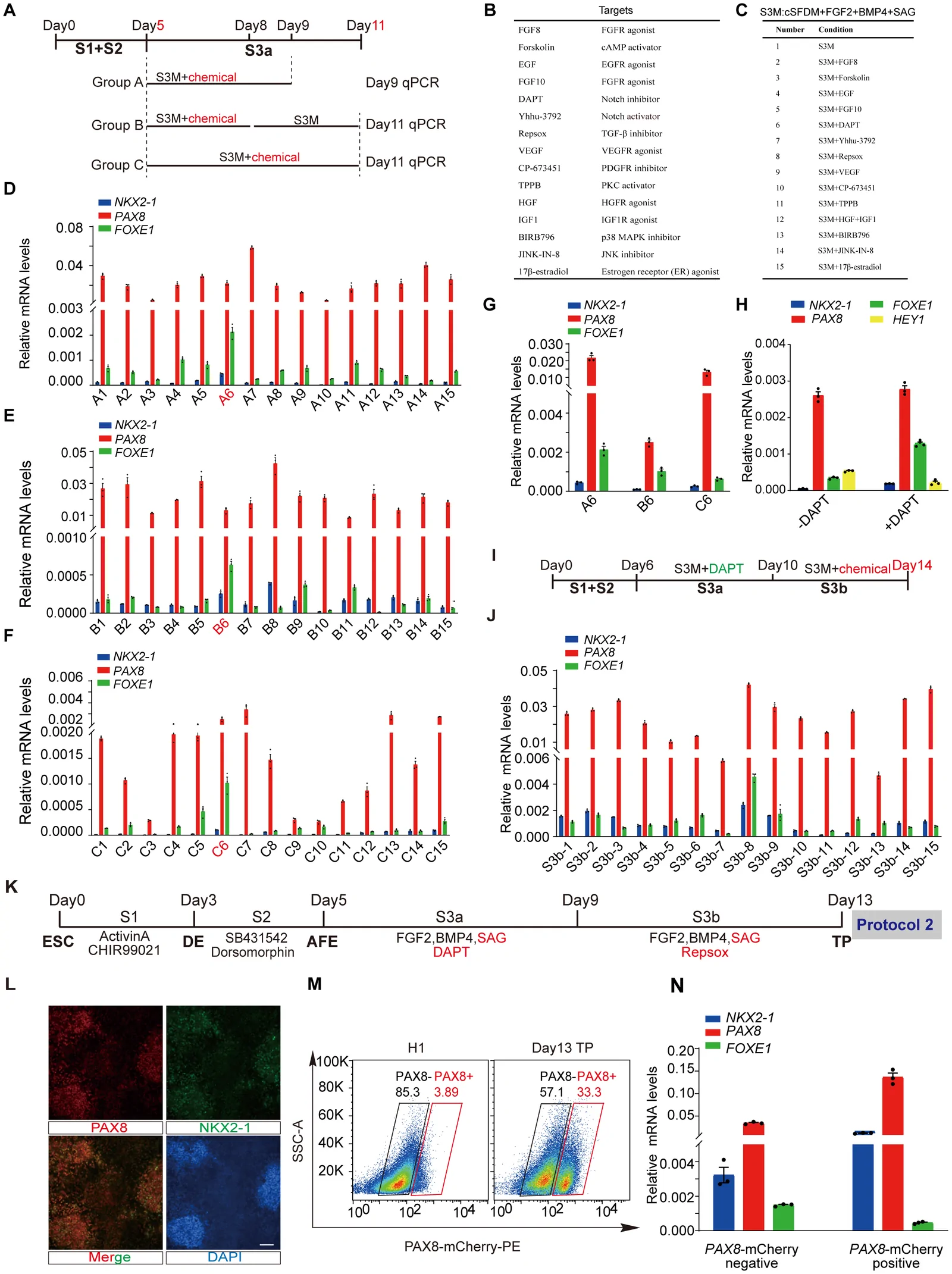
Systematic small-molecule screening and temporal pathway modulation establish Protocol 2 for enhanced thyroid progenitor induction. **(A)** Schematic illustrating the temporal dissection of Stage 3 into discrete S3a and S3b windows for stepwise pathway modulation during thyroid progenitor induction. **(B, C)** Matrix of the 15 small-molecule combinations screened across the S3a and S3b windows. **(D–F)** RT-qPCR time-course analyses evaluating the effect of varying DAPT treatment duration during S3a on the expression of *NKX2-1, PAX8*, and *FOXE1*. Data represent mean ± SD (n = 3). **(G)** RT-qPCR analysis comparing *FOXE1* induction efficiency under different S3a treatment paradigms, revealing the optimal DAPT exposure window for *FOXE1* activation. **(H)** RT-qPCR analysis of *NKX2-1*, *PAX8*, *FOXE1*, and *HEY1* in the absence (−DAPT) or presence (+DAPT) of DAPT. Data are presented as mean ± SD (n = 3). **(I)** Design framework of the refined differentiation protocol derived from the screening data, outlining the combinatorial logic for S3a and S3b factor integration. **(J)** RT-qPCR validation of the top-performing S3b condition on Day 13 thyroid progenitors, demonstrating synergistic upregulation of *NKX2-1*, *PAX8*, and *FOXE1* by sequential DAPT and RepSox treatment. Data represent mean ± SD (n = 3). **(K)** Schematic of Protocol 2 derived from systematic pathway screening, incorporating DAPT (Notch inhibitor) in S3a and RepSox (TGF-β inhibitor) in S3b. **(L)** Immunofluorescence of Day 14 Protocol 2 progenitors, confirming robust co-expression of NKX2–1 and PAX8 at the protein level. Scale bar, 100 μm. **(M)** Representative FACS plot used to isolate *PAX8*-mCherry^-^ and *PAX8*-mCherry^+^ populations from Day 13 Protocol 2 progenitors. **(N)** RT-qPCR analysis of the sorted populations for *NKX2-1*, *PAX8*, and *FOXE1* transcripts. Data represent mean ± SD (n = 3).

To identify alternative potent upstream regulators, we performed a systematic screen of 15 small-molecule combinations (**Figures 2B, C**). By partitioning S3 into discrete temporal windows (**Supplementary Materials 3a, b**), we identified the Notch inhibitor DAPT as a critical driver of lineage specification during the S3a phase (**Figures 2A**, **D–G**). Consistent with on-target Notch inhibition, DAPT treatment downregulated the canonical Notch target *HEY1* while concomitantly increasing *NKX2-1*, *PAX8*, and *FOXE1* transcripts (**Figure 2H**), confirming that the Notch pathway is active in this differentiation system and is functionally modulated by DAPT (30). Further optimization demonstrated that sequential treatment with the TGF-β inhibitor RepSox during S3b acted synergistically with DAPT to markedly upregulate thyroid lineage markers (**Figures 2I, J**), thereby establishing the methodological framework for Protocol 2 (**Figure 2K**).

**Figure 3:**
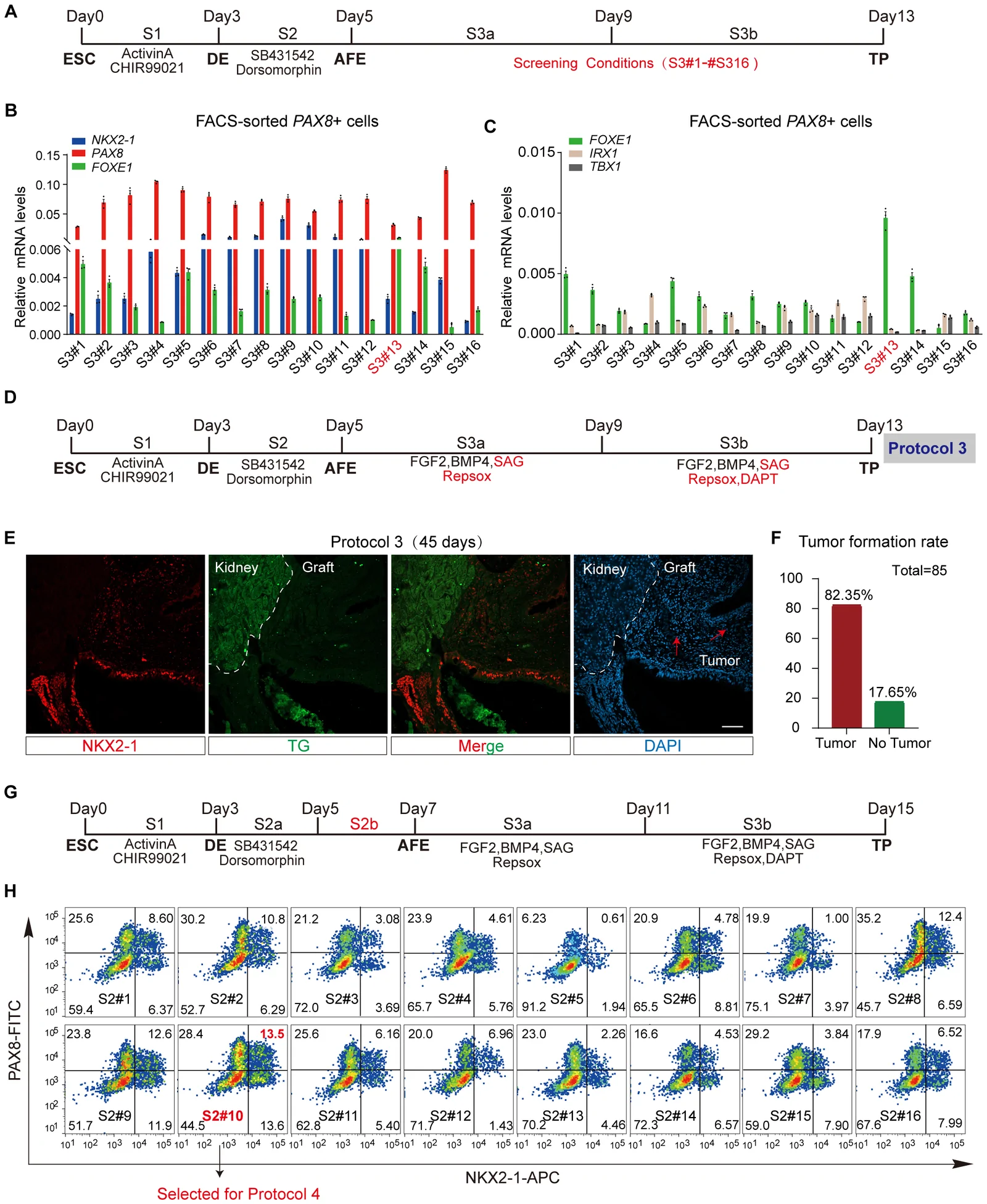
*In vivo* tumorigenicity of Protocol 3 uncovers lineage bottlenecks and directs Stage 2 chemical refinement for Protocol 4. **(A)** Schematic of the staged screening strategy. Stage 3 was temporally dissected into S3a (days 5–9) and S3b (days 9–13) windows for independent combinatorial pathway modulation during thyroid progenitor specification. **(B)** RT-qPCR screen of *NKX2-1*, *PAX8*, and *FOXE1* in FACS-purified PAX8^+^ cells from 16 S3a/S3b combinations. Data represent mean ± SD (n = 3). **(C)** RT-qPCR-based profiling of non-thyroid lineage markers (e.g., *IRX1, TBX1*) across the 16 conditions, confirming that the selected regimen maintains minimal non-thyroid lineage expression. Data represent mean ± SD (n = 3). **(D)** Refined Protocol 3, extending RepSox to S3a and combining it with DAPT. **(E)** Immunofluorescence of Protocol 3-derived grafts at Day 45. Yellow arrows indicate TG-positive regions; Red arrows indicate tumor regions. Tumors were defined as described in the main text. Scale bars, 100 μm. **(F)** Tumor formation rate in mice transplanted with Protocol 1–3 progenitors (n = 85 grafts). The bar graph shows 82.35% (70/85) developed tumors and 17.65% (15/85) remained tumor-free. **(G)** Schematic of the Stage 2 (S2) screening strategy. S2 is subdivided into S2a and S2b windows for systematic combinatorial modulation. **(H)** Representative FACS plots of NKX2–1 and PAX8 induction efficiency. Flow cytometry analysis of PAX8 and NKX2–1 expression across the 16-condition S2 screen (#1–#16). Condition #10 shows the highest efficiency of PAX8^+^/NKX2-1^+^ double-positive cells (highlighted in red, 13.5%).

While Protocol 2 increased overall *FOXE1* transcript levels and achieved robust NKX2–1 and PAX8 co-expression at the protein level by Day 13 (**Figure 2L**), these progenitors failed to undergo terminal functional maturation upon *in vivo* transplantation. To elucidate the underlying cause of this maturation arrest, we performed downstream molecular analysis on FACS-isolated populations (**Figure 2M**), which unveiled a notable transcriptional discrepancy. Although *NKX2–1* and *PAX8* transcripts were enriched within the sorted *PAX8*-mCherry^+^ fraction, *FOXE1* mRNA was unexpectedly downregulated in this target fraction and predominantly restricted to the *PAX8*-mCherry^-^ population (**Figure 2N**). Collectively, these findings indicate that while the DAPT/RepSox axis successfully induces *FOXE1*, it introduces a lineage bias that uncouples *FOXE1* expression from the target PAX8^+^ progenitor pool. This persistent transcriptional dissociation likely accounts for the *in vivo* developmental failure, necessitating further optimization.

### An optimized combinatorial approach generates *FOXE1*-expressing thyroid progenitors and directs upstream refinement

Building upon insights from our Protocol 2 screening, we observed that while DAPT treatment effectively induced FOXE1, it concomitantly introduced a lineage bias, culminating in FOXE1 expression predominantly within the PAX8-negative population. To uncouple FOXE1 induction from this lineage deviation, we hypothesized that the temporal window of DAPT application should be delayed to a later phase of differentiation (post-PAX8 specification), while the administration of RepSox could be advanced. Based on this rationale, we designed a combinatorial matrix of 16 small-molecule combinations across the S3a and S3b windows (**Figure 3A**; **Supplementary Figure 3A**). Utilizing a *PAX8*-mCherry reporter line, we isolated the PAX8^+^ progenitor population via fluorescence-activated cell sorting (FACS) and systematically profiled them via RT-qPCR. We identified synergistic conditions driving robust *NKX2-1*, *PAX8*, and *FOXE1* co-expression (**Figure 3B**). Notably, the optimal condition also displayed minimal non-thyroid endodermal marker expression (**Figure 3C**), thereby confirming its lineage fidelity. This dual-filtering approach culminated in a refined combinatorial strategy designated as Protocol 3 (**Figure 3D**).

While Protocol 3 effectively orchestrated the terminal transcriptional program, enabling *in vivo* thyroglobulin (TG) expression upon transplantation (**Figure 3E**), it failed to overcome a major translational roadblock: the inherent tumorigenicity characteristic of early hPSC derivatives. Indeed, consistent with earlier iterations (Protocols 1 and 2), the accumulated historical cohort of these Stage 2-unoptimized grafts (Protocols 1–3) exhibited a high cumulative tumor incidence rate of 82.35% (70/85 grafts) (**Figure 3F**)—a neoplastic propensity uniformly observed regardless of Stage 3 refinements (**Supplementary Figure 3B**). These *in vivo* findings strongly indicated that while Stage 3 manipulations correctly coordinate late-stage maturation, the baseline tumorigenic risk stems from incomplete upstream lineage specification. Consequently, we hypothesized that further developmental refinement during the anterior foregut endoderm stage (Stage 2) was imperative. We subdivided Stage 2 into S2a and S2b windows and conducted an additional systematic 16-condition screen targeting the S2b phase. The design of this S2b combinatorial matrix was predicated on advancing the temporal application of key small molecules that had demonstrated efficacy during the S3 optimization phase, exposing earlier progenitors to these pathway modulators (**Figure 3G**; **Supplementary Figure 3C**). To accurately assess the induction efficiency of these upstream regimens, we performed intracellular flow cytometry. By implementing a stringent gating strategy and utilizing robust negative controls to verify staining specificity (**Supplementary Figure 3D**), we precisely quantified PAX8 and NKX2–1 co-expression, ultimately identifying Condition #10 as the optimal S2 protocol for downstream integration (**Figure 3H**).

### Precise modulation of anterior foregut specification attenuates tumorigenicity and enables early *in vivo* maturation

**Figure 4:**
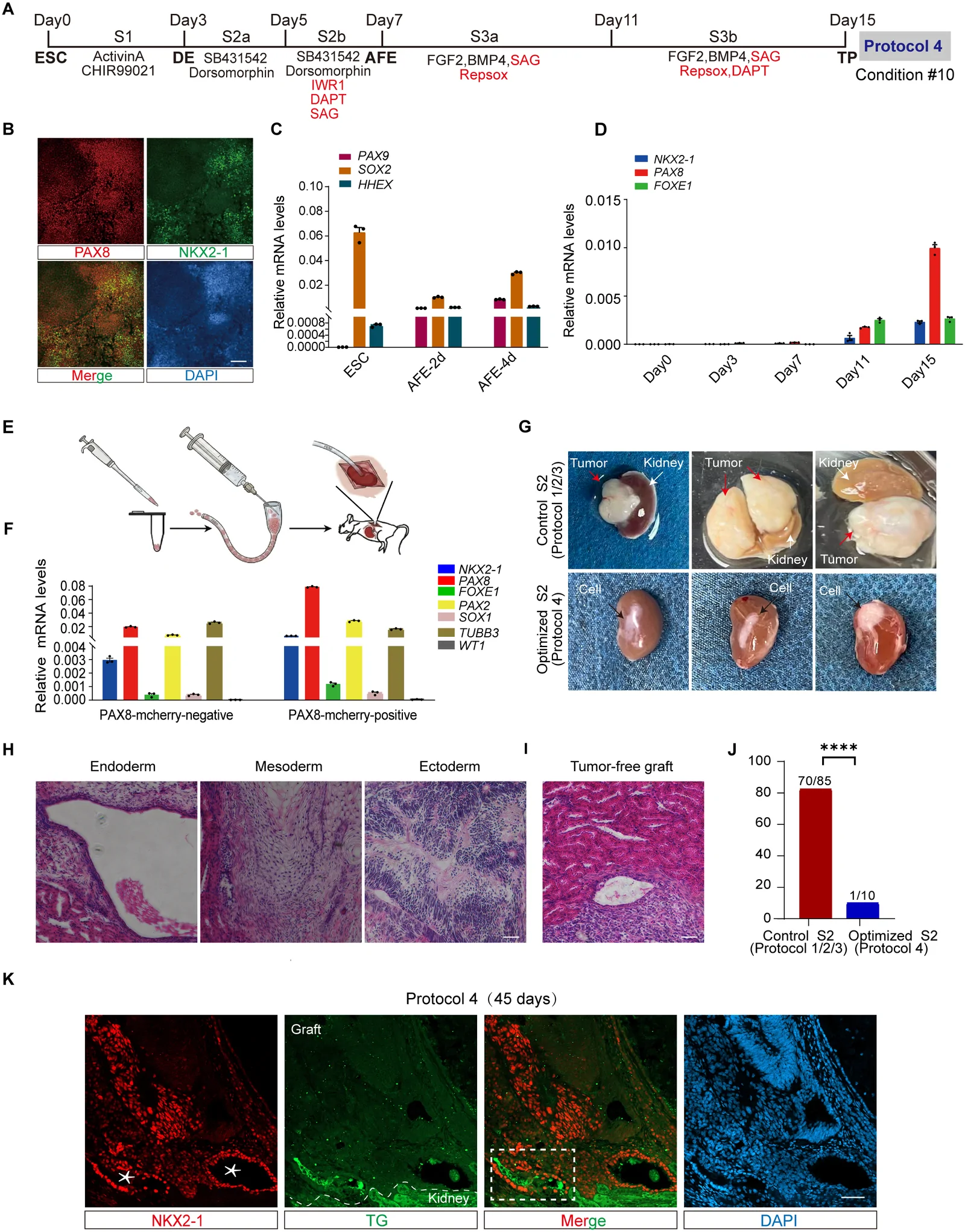
Protocol 4 attenuates graft tumorigenicity and supports early *in vivo* morphogenesis of thyroid follicle-like structures. **(A)** Final optimized three-stage differentiation regimen (Protocol 4). **(B)** Immunofluorescence of unsorted Day 15 Protocol 4 cells demonstrating co-expression of NKX2–1 and PAX8 within the target progenitor subpopulation. Scale bars, 100 μm. **(C)** RT-qPCR analysis of anterior foregut endoderm markers (*PAX9*, *SOX2*, and *HHEX*) in H1, AFE-2d, and AFE-4d cells. Data represent mean ± SD (n = 3). **(D)** Time-course RT-qPCR analysis of thyroid lineage markers (*NKX2-1*, *PAX8*, and *FOXE1*) across the full Protocol 4 differentiation trajectory. Data represent mean ± SD (n = 3). **(E)** Diagram of the kidney subcapsular transplantation procedure in immunodeficient mice. **(F)** RT-qPCR analysis of *NKX2-1*, *PAX8*, *FOXE1*, *PAX2*, *SOX1*, *TUBB3*, and *WT1* in *PAX8*-mCherry-negative and *PAX8*-mCherry-positive cells. Data represent mean ± SD (n = 3). **(G)** Morphology of kidney grafts at 45 days post-transplantation. Upper panel (Control S2, Protocol 1–3): Tumor-bearing kidneys. White arrows indicate host kidney; red arrows indicate large tumor masses. Lower panel (Optimized S2, Protocol 4): Tumor-free kidneys. Black arrows indicate the grafted cell region, which shows no visible tumor formation. **(H)** Representative hematoxylin and eosin (H&E) staining images of teratoma tissues derived from control grafts (Protocol 1–3), displaying structures from all three germ layers: endoderm, mesoderm, and ectoderm. Scale bar, 100 μm. **(I)** Representative H&E staining image of a tumor-free graft in the kidney capsule from the optimized protocol (Protocol 4), showing normal tissue architecture without teratoma formation. Scale bar, 100 μm. **(J)** Quantitative comparison of tumor formation rates between control S2 (82.35%, 70/85 grafts) and optimized S2 conditions (10%, 1/10 grafts). Statistical significance was calculated using Fisher’s exact test. ****P < 0.0001. **(K)** Graft analysis at 45 days post-transplantation. Single-channel images (upper) and enlarged merges (lower) of TG/NKX2-1. Dashed line: graft/kidney boundary; asterisks (*): regenerated follicular lumens; dashed box: basolateral deposition of TG. Scale bars, 50 μm.

Hypothesizing that the persistent tumorigenic risk and maturation arrest of hPSC-derived progenitors stem from incomplete lineage specification during the early anterior foregut endoderm (AFE) induction phase, we systematically dissected the Stage 2 (S2) temporal window using a 16-condition screen (**Figure 3G**; **Supplementary Figure 3C**). By interrogating the dynamics of the Notch, Hedgehog, Wnt, and retinoic acid pathways, we identified a precise combinatorial regimen—integrating IWR1 (a Wnt inhibitor), DAPT, and SAG (a Smoothened agonist)—that maximized the synchronized induction of NKX2–1 and PAX8 (**Figure 3H**), which established the cornerstone of our finalized Protocol 4 (**Figure 4A**). Immunofluorescence staining confirmed detectable co-expression of the core thyroid transcription factors within a subset of the Protocol 4-derived Day 15 cells (**Figure 4B**). During embryonic development, the AFE serves as a critical developmental gateway through which pluripotent progenitors transition to committed foregut lineages; insufficient time at this checkpoint may leave a residual fraction of cells incompletely specified and thus prone to neoplastic divergence upon transplantation. We therefore extended the AFE induction window from 2 to 4 days to provide a longer developmental window for Notch, Hedgehog, and Wnt pathway modulation to reinforce lineage commitment. Importantly, this extension did not compromise anterior foregut identity: RT-qPCR showed that AFE markers (*PAX9, SOX2*, and *HHEX*) were appropriately expressed in both AFE-2d and AFE-4d cells relative to the H1 baseline (**Figure 4C**). A time-course RT-qPCR analysis across the full differentiation trajectory further confirmed the progressive and synchronized activation of thyroid lineage transcripts (*NKX2-1*, *PAX8*, and *FOXE1*) under Protocol 4 (**Figure 4D**).

This early developmental refinement proved to be a critical optimization step for both lineage fidelity and graft safety. Prior to transplantation beneath the kidney capsule of immunodeficient mice (**Figure 4E**), the identity of the sorted *PAX8-*mCherry^+^ population was characterized by RT-qPCR. This analysis demonstrated a robust enrichment of core thyroid markers (*NKX2-1*, *PAX8*, *FOXE1*) relative to the *PAX8*-mCherry^-^ fraction. In contrast, non-thyroid lineage markers exhibited a more nuanced pattern: slight elevations in *PAX2* and *SOX1* suggested minor off-target commitment (such as toward renal cell fates) (31), whereas unchanged levels of *TUBB3* and *WT1* ruled out broad neural or mesothelial diversion (**Figure 4F**). Consequently, the PAX8^+^/NKX2-1^-^ subset, which constitutes a substantial proportion of the sorted population, represents a heterogeneous pool where only a minor fraction exhibits distinct off-target commitment, rather than representing a mass diversion to a single discrete lineage. Optimization of S2 signaling markedly reduced the neoplastic potential inherent to earlier protocols. In direct comparison with the combined historical controls (Protocols 1–3), the tumor incidence rate markedly decreased from 82.35% (70/85 grafts) to 10% (1/10 grafts) in the Protocol 4 cohort, with no overt teratoma-like growth observed (**Figures 4G, J**; Fisher’s exact test, *P* < 0.0001). This substantial reduction was corroborated at the histological level: hematoxylin and eosin (H&E) staining of control grafts revealed overt teratomatous tissue containing derivatives of all three germ layers, whereas grafts from the optimized Protocol 4 displayed organized, non-teratomatous tissue in which follicle-like structures were discernible (**Figures 4H, I**).

Beyond this improved *in vivo* safety profile, Protocol 4-derived grafts demonstrated enhanced potential for *in vivo* structural organization, characterized by the formation of well-organized NKX2-1^+^ thyroid follicles with luminal TG accumulation by Day 45 post-transplantation (**Figure 4K**). Collectively, these results indicate that precise spatiotemporal signaling modulation during the AFE stage is a critical determinant of reduced tumorigenicity and enables the early *in vivo* morphogenesis of hPSC-derived thyroid progenitors; however, definitive functional maturation remains to be demonstrated.

## Discussion

In this study, we established a systematically optimized differentiation paradigm that efficiently generates thyroid progenitors from human pluripotent stem cells (hPSCs), and characterized the stage-specific signaling requirements underlying this optimization. By dissecting these requirements, our results indicate that the synchronized induction of NKX2-1, PAX8, and FOXE1 is associated with a critical developmental checkpoint for subsequent *in vivo* and terminal functional maturation. Importantly, our findings indicate that precise signal modulation during the anterior foregut endoderm (AFE) stage is the critical determinant in mitigating inherent tumorigenic risks, effectively safeguarding lineage commitment by reducing residual undifferentiated populations. Ultimately, this paradigm reconciles efficient lineage specification with a markedly improved safety profile. While our *in vivo* transplantation data show that these optimized progenitors engraft and display early maturation features (e.g., follicular organization and luminal TG accumulation), these observations remain descriptive. Definitive functional efficacy—including *de novo* thyroid hormone synthesis at physiologically meaningful levels and the capacity to correct hypothyroidism—remains to be established in future studies.

A key feature of this protocol is the transcriptional activation and synchronized co-expression of *FOXE1* alongside *NKX2–1* and *PAX8*. In our hands, conventional directed differentiation protocols applied to the H1 hESC line consistently yielded insufficient *FOXE1* expression, posing a major obstacle to lineage transition. This limitation is particularly intriguing in light of recent findings by Undeutsch et al. (16), who achieved *FOXE1* expression during human iPSC specification but likewise found thyroid differentiation to be highly protocol-dependent. The specific bottlenecks—*FOXE1* induction in our system versus sodium/iodide symporter (NIS) expression and T4 secretion in theirs—vary depending on the starting cell line and culture conditions. In murine models, *Foxe1* is dispensable for initial endodermal specification but essential for thyroid primordium migration (22, 32), directly transactivating *Tg*, *Tpo*, and *Nis* in mature thyrocytes (33). Our findings provide correlative evidence that *FOXE1* is associated with advanced morphological and early *in vivo* maturation: when *FOXE1* induction was inadequate (Protocol 1), grafts failed to form TG^+^ follicular structures; conversely, synchronized *NKX2-1/PAX8/FOXE1* co-induction (Protocol 4) was associated with organized follicular morphogenesis and luminal TG accumulation. Importantly, recent literature provides the direct genetic proof our study lacks: Fonseca et al. recently demonstrated via a *Foxe1*-deficient mouse ESC-derived organoid model that *Foxe1* deficiency impairs thyroid fate determination (23). While this external evidence strongly supports our observations, definitive proof of FOXE1’s absolute essentiality within our specific step-wise system still requires direct genetic validation (e.g., FOXE1 knockout or knockdown) in future studies.

Beyond functional efficacy, tumorigenicity remains a formidable bottleneck impeding the clinical translation of hPSC-derived cellular therapies. Existing teratoma-mitigation efforts primarily rely on downstream, post-differentiation purging—such as FACS/MACS sorting for SSEA-4/TRA-1-60, the integration of inducible suicide genes, or the application of selective cytotoxic small molecules (34, 35).

However, these retrospective clearance strategies are frequently compromised by high manufacturing costs, substantial cell loss, and potential genomic instability. In contrast, we introduce an upstream, developmentally grounded preventive paradigm. We propose that precise signaling modulation at the AFE stage curtails tumorigenic risk by reinforcing a developmental checkpoint that promotes lineage commitment and minimizes the residual undifferentiated cell fraction prior to transplantation. Crucially, because the AFE constitutes a highly conserved embryonic gateway shared by multiple endodermal derivatives (including the lung, thymus, esophagus, and parathyroid), this upstream quality-control strategy holds broad generalizability. It serves as a robust framework for systematically reducing oncogenic risk across other foregut-derived lineages, thereby improving the translational safety profile.

Several limitations within the current study must be acknowledged. First, transplantation was performed in euthyroid mice with basal thyroid-stimulating hormone (TSH) levels, providing a suboptimal trophic stimulus for graft maturation, and graft function was not assessed by circulating hormone measurements in the present study. Indeed, previous studies transplanting fully differentiated thyroid follicles into strongly TSH-driven hypothyroid mice still required at least 5 weeks for functional integration (36). Therefore, rigorous evaluation of graft function in future studies will require a hypothyroid recipient model coupled with comprehensive systemic assessments—including measurements of circulating hormones (T4, triiodothyronine (T3), TSH), iodide uptake, and TSH-stimulated T4 secretion (14, 36–38). Second, graft characterization was limited to NKX2–1 and TG immunostaining; quantitative analysis of follicular organization, engraftment efficiency, and the expression of terminal maturation markers, such as thyroid stimulating hormone receptor (TSHR), sodium/iodide symporter (NIS/SLC5A5), and thyroid peroxidase (TPO), is still required.

Beyond *in vivo* functional assays, further optimization is needed to refine safety profiles and methodological frameworks. Third, although tumorigenicity was markedly reduced from 82.35% to 10%, this residual risk must be entirely eradicated prior to clinical translation (17). Finally, regarding our *in vitro* maturation step, we adopted an air–liquid interface (ALI) 3D culture rather than a Matrigel-embedded (MTG) organoid format. While the ALI configuration provides direct apical gas exchange and a defined basal supply of maturation factors—favoring the apico-basal epithelial polarization prerequisite for follicular lumen formation without the batch-to-batch variability of Matrigel—a direct head-to-head comparison of ALI versus MTG was not performed (39). Evaluating these platforms, alongside exploring orthotopic or large-animal models for long-term graft function and hypothalamic-pituitary-thyroid (HPT) axis integration, represent crucial directions for future optimization. Furthermore, as an exploratory preclinical study, the sample sizes employed here were determined based on practical feasibility and biological precedent rather than formal power calculations, an approach consistent with general recommendations for justifying sample sizes in exploratory studies (40).

In conclusion, we have developed a systematically optimized three-stage framework that directly addresses two major challenges in hPSC-based thyroid regeneration: insufficient *FOXE1* induction and the high tumorigenic risk of hPSC-derived grafts. By identifying the AFE stage as a critical window for safety modulation and demonstrating FOXE1’s association with maturation, this study provides an efficient technical route for generating low-tumorigenicity thyroid progenitors capable of early *in vivo* morphogenesis. Concomitantly, it establishes a robust platform for modeling human thyroid development. Achieving definitive functional maturation and demonstrating therapeutic efficacy in hypothyroid models remain clearly defined objectives for our future work.

## Materials and methods

### Mice

Male BALB/cNj-*Foxn1*^nu^/Gpt (c-Nude) mice, aged 6–8 weeks, were purchased from GemPharmatech (Nanjing, China). The mice were acclimated and maintained under controlled, specific pathogen-free (SPF) environmental conditions with a 12-h light/12-h dark cycle. The animal room temperature was regulated between 18–24 °C, and the relative humidity was maintained at 40–60%. All animal care and experimental procedures were conducted in strict accordance with the guidelines for the care and use of laboratory animals and were formally approved by the Institutional Animal Care and Use Committee (IACUC) of Hunan University. Mice within this age range were sequentially allocated for subsequent surgical procedures. A total of 95 mice were used, with one graft transplanted per animal: 10 mice were assigned to the Protocol 4 (optimized S2) group, and 85 mice were used across Protocols 1–3, which serve as accumulated historical controls for the unoptimized S2 condition.

### Human Pluripotent Stem Cell (hPSC) maintenance

The human embryonic stem cell (hESC) line H1 was kindly provided by Dr. Kai Wang (School of Basic Medical Sciences, Peking University Health Science Center). The cells were maintained on Matrigel-coated (Corning) plates in mTeSR1 medium (STEMCELL Technologies) at 37 °C in a humidified incubator with 5% CO₂. For routine culture, cells were passaged using Accutase (STEMCELL Technologies) every 4–5 days and seeded onto fresh Matrigel-coated plates at a density of 1–2 × 10^4^ cells/cm². To enhance cell survival, the mTeSR1 medium was supplemented with 10 μM Y-27632 (a Rho-associated protein kinase inhibitor) for the first 24 h post-passaging.

### Generation of *PAX8*-mCherry reporter line

A P2A-mCherry cassette was knocked in immediately upstream of the *PAX8* stop codon to generate a transcriptional reporter. The targeting strategy involved CRISPR/Cas9-mediated homology-directed repair (HDR). The specific guide RNA (sgRNA) targeting the *PAX8* locus (5′-CGGCCTTTGACCATCTGTAG-3′) was cloned into the pSpCas9(BB)-2A-Puro vector. A donor targeting vector was constructed containing the P2A-mCherry-loxP-Puro-loxP sequence flanked by a 1.2 kb left homology arm and a 1.1 kb right homology arm. hESCs were co-electroporated with the Cas9-sgRNA plasmid and the donor vector, followed by puromycin selection. Correct integration was validated by genomic PCR and Sanger sequencing. The selection cassette was subsequently excised by transient expression of Cre recombinase to yield a transgene-free reporter line. Reporter fidelity was confirmed by the co-localization of mCherry fluorescence with endogenous *PAX8* expression, as detected by immunofluorescence, during thyroid-directed differentiation.

### Thyroid progenitor differentiation protocol

#### Stage 1: Definitive endoderm induction (Days 0–3)

H1 human embryonic stem cells (hESCs) were dissociated using TrypLE and seeded at a density of 1.05 × 10^6^ cells/well onto Matrigel-coated 6-well plates in mTeSR1 medium supplemented with 10 μM Y-27632. Upon reaching approximately 40% confluency (~18 h), the cells were washed three times with DMEM/F12 and replenished with fresh mTeSR1. Directed differentiation was initiated when cells reached 70–80% confluency (approximately 24 h later). On Day 1, cells were washed twice with pre-warmed DMEM/F12 and cultured in MS12 medium (composition listed in **Supplementary Table 1**) supplemented with 100 ng/mL Activin A (a TGF-β pathway activator) and 3 μM CHIR99021 (a Wnt pathway activator). After 30–33 h of incubation (Day 2), the cells were washed twice to remove CHIR99021 and subsequently cultured in MS12 medium containing 100 ng/mL Activin A. On Day 3, the cells were rinsed once and maintained in fresh MS12 medium with 100 ng/mL Activin A for an additional 24 h.

#### Stage 2: Induction of anterior foregut endoderm

Stage 2 differentiation was performed in complete chemically defined serum-free medium (cSFDM; formulation detailed in **Supplementary Table 2**). On Day 3, definitive endoderm (DE) cells were carefully rinsed twice with DMEM/F12; extreme care must be taken during this step as the cell monolayer is highly susceptible to detachment. The cells were then incubated with Gentle Cell Dissociation Reagent (GCDR; STEMCELL Technologies) at room temperature for 8–10 min to dissociate them into small cell clumps. After aspirating the GCDR, 1–5 mL of DMEM/F12 was added, and the cell clumps were gently detached using a cell scraper and transferred into a 15 mL conical tube without centrifugation to preserve clump integrity. The cell clump suspension was plated onto Matrigel-coated plates at a 1:2 to 1:6 split ratio in cSFDM supplemented with 10 μM SB431542 (a TGF-β inhibitor) and 2 μM Dorsomorphin (a BMP inhibitor).

The duration and specific treatment of Stage 2 varied depending on the protocol iterations:

- For Protocols 1–3: Cells were maintained in this medium until Day 5 to yield anterior foregut endoderm (AFE).
- For Protocol 4 (Optimized): This initial phase (designated as S2a) was maintained until Day 5. Subsequently, cells were switched to an S2b medium consisting of cSFDM supplemented with 10 μM SB431542, 2 μM Dorsomorphin, 3 μM IWR1, 10 μM DAPT, and 0.5 μM SAG, and cultured until Day 7 to yield AFE.

#### Stage 3: Specification of thyroid progenitors

The temporal window and specific cytokine combinations of Stage 3 were iteratively optimized. Following the completion of Stage 2 (Day 5 for Protocols 1–3; Day 7 for Protocol 4), AFE cells were gently rinsed twice with DMEM/F12 and cultured in varying conditions based on cSFDM supplemented with core factors (100 ng/mL FGF2 and 100 ng/mL BMP4). The culture medium was replenished every two days.

- Protocol 1: Stage 3 spanned from Days 5**–**15, during which cells were continuously cultured in cSFDM supplemented with FGF2 and BMP4.
- Protocol 2: Stage 3 spanned from Days 5–13 and was subdivided into two phases: S3a (Days 5–9) utilizing FGF2, BMP4, 0.5 μM SAG, and 5 μM DAPT; followed by S3b (Days 9–13) utilizing FGF2, BMP4, 0.5 μM SAG, and 10 μM RepSox.
- Protocol 3: Stage 3 spanned from Days 5**–**13, subdivided into S3a (Days 5–9) utilizing FGF2, BMP4, 0.5 μM SAG, and 10 μM RepSox; and S3b (Days 9–13) utilizing FGF2, BMP4, 0.5 μM SAG, 10 μM RepSox, and 5 μM DAPT.
- Protocol 4 (Optimized): The Stage 3 temporal window was adjusted to span from Days 7**–**15. Cells were cultured in S3a medium (Days 7–11) consisting of FGF2, BMP4, 0.5 μM SAG, and 10 μM RepSox. On Day 11, cells were transitioned to S3b medium (Days 11–15) consisting of FGF2, BMP4, 0.5 μM SAG, 10 μM RepSox, and 5 μM DAPT.

At the end of Stage 3 (Day 15 for Protocols 1 and 4; Day 13 for Protocols 2 and 3), the resulting cells were harvested as hPSC-derived thyroid progenitors (TPs) for subsequent analyses or applications.

### Gene expression analysis

Total RNA was extracted using the RNA-easy Isolation Reagent (Vazyme, China, Cat# R701-01). Reverse transcription was performed using HiScript II QRT SuperMix for qPCR (+gDNA wiper) (Vazyme, China, Cat# R223-01) according to the manufacturer’s protocol. Quantitative reverse transcription PCR (RT-qPCR) was performed using ChamQ Universal SYBR qPCR Master Mix (Vazyme, China, Cat# Q711-02) on a QuantStudio 1 Real-Time PCR System. Gene expression levels were calculated using the ^2-ΔΔCt^ method and normalized to *GAPDH* as an internal control. Specific primer sets were designed using Primer Premier software to span exon–exon junctions, thereby avoiding the amplification of genomic DNA. All primer sequences used in this study are listed in **Supplementary Table 3**.

### Air-Liquid Interface (ALI) culture for *in vitro* maturation

To induce the *in vitro* maturation of thyroid progenitors, Day 15 Stage-3 (S3) thyroid progenitor cells were subjected to a 3D air-liquid interface (ALI) culture system. Briefly, the cells were rinsed with PBS and incubated with Accutase (STEMCELL Technologies) for 10–15 min at 37 °C to obtain single-cell suspensions. Following dissociation, the cells were rinsed twice with DMEM/F12 and centrifuged at 300 g for 3 min. The resulting cell pellet was resuspended in aggregation medium consisting of complete chemically defined serum-free medium (cSFDM) supplemented with 100 ng/mL FGF2, 100 ng/mL BMP4, and 10 μM Y-27632. Subsequently, cell suspensions containing 0.3 million cells were seeded into each well of a V-bottom 96-well plate and centrifuged at 300 g for 3 min. This specific seeding density was chosen based on our previously established protocol for generating robust 3D progenitor clusters (27). Consistent with this established framework, densities within the range of 0.1–0.4 million cells per V-bottom well were validated to optimally support compact aggregate formation, ensure reliable cell-cell adhesion, and maintain cell viability (e.g., preventing central necrosis) throughout the 16-hour aggregation period. The plates were then incubated at 37 °C for 16 h to allow for the spontaneous formation of 3D thyroid progenitor clusters. The assembled progenitor clusters were collected, rinsed twice with DMEM/F12, and loaded onto 12-well ALI Transwell inserts (LABSELECT, Cat# 14212). For the ALI culture, approximately 750 μL of maturation medium was added to the lower chamber of each well. The combinatorial condition matrix for the specific small-molecule combinations is detailed in **Supplementary Figure 1C**. The final concentrations of supplements and small molecules used in the basic media and differentiation conditions were as follows: forskolin (FSK, 20 μM), 3-isobutyl-1-methylxanthine (IBMX, 0.1 mM), EGF (20 ng/mL), TSH (1 mU/mL), dexamethasone (Dexa, 50 nM), SB431542 (10 μM), NaI (10 μM), FGF2 (20 ng/mL), FGF10 (20 ng/mL), Heparin (100 ng/mL), IGF1 (50 ng/mL), GlutaMAX (1×), non-essential amino acids (NEAA, 1×), Vitamin C (50 ng/mL), β-Mercaptoethanol (0.055 mM), Sodium pyruvate (1×), penicillin-streptomycin (PS, 1×), BSA (0.25%), and BaCl_2_ (0.8 mM). The cells were maintained under these maturation conditions for 23 days, with the medium replenished every other day.

### Immunofluorescence and imaging for cell culture

The cells were washed twice in PBS and fixed with 4% paraformaldehyde for 15 min at room temperature. After fixation, the cells were blocked in blocking buffer (PBS containing 0.2% Triton X-100 and 0.5% normal donkey serum (Solarbio)) for 1 h at room temperature. Primary antibodies diluted in PBST (PBS containing 0.2% Triton X-100) were applied and incubated overnight at 4 °C. After three washes with PBST, the cells were incubated with secondary antibodies and DAPI (Solarbio) for 1 h at room temperature. All antibodies used in this study are listed in **Supplementary Table 4**. Finally, the cells were washed three times with PBS prior to imaging. Image acquisition was conducted using a Zeiss LSM 980 confocal microscope, and the images were processed using Photoshop or Zeiss Zen.

### Flow cytometry and cell sorting

The cells were washed with PBS and dissociated by incubation with Accutase (STEMCELL Technologies) at 37 °C for 1–3 min. The enzymatic reaction was quenched by dilution with DMEM/F12, followed by two additional washes with PBS. For intracellular staining, cells were fixed using 200 μL of BD Cytofix/Cytoperm™ Buffer (BD Biosciences) at 4 °C for 20 min and subsequently washed three times with BD Perm/Wash™ Buffer (BD Biosciences). The fixed cells were then incubated overnight at 4 °C with 50 μL of primary antibody diluted in Perm/Wash Buffer. Secondary antibody-only controls (cells processed through fixation and permeabilization without primary antibody, but incubated with secondary antibody) were included to establish gating thresholds and account for non-specific background binding. After two washes with Perm/Wash Buffer, the cells were incubated for 2 h with fluorescently labelled secondary antibodies. Following two final washes in Perm/Wash Buffer, the stained cells were analyzed. For cell surface staining, single-cell suspensions were incubated with 50–100 μL of antibody diluted in PBS at 4 °C for 1 h and washed twice with PBS prior to sorting. All antibodies used in this study are listed in **Supplementary Table 4**. All samples were analyzed using a flow cytometer, and the data were processed using FlowJo software.

### Kidney capsule transplantation and immunofluorescence analysis

For *in vivo* functional assessment and tumorigenicity evaluation, thyroid progenitors generated from the evaluated differentiation protocols were utilized. Specifically, cells were harvested on Day 15 for Protocols 1 and 4, and on Day 13 for Protocols 2 and 3. Prior to transplantation, the cells were purified using fluorescence-activated cell sorting (FACS) to isolate the entire *PAX8*-mCherry^+^ progenitor population. The sorted *PAX8*-mCherry^+^ cells were resuspended in DMEM/F12. Nude mice were anesthetized with 2% isoflurane inhalation, and the purified TPs were transplanted into the subcapsular space of the left kidney using PE50 tubing. Following the surgical procedure, the wound was disinfected with povidone-iodine to prevent infection. The mice were then placed on a heating pad to maintain body temperature and closely monitored until fully recovered from anesthesia to ensure optimal post-operative welfare and minimize distress. At 45 and 90 days post-transplantation, the mice were euthanized by cervical dislocation, performed by trained personnel in strict accordance with the protocols approved by the Institutional Animal Care and Use Committee (IACUC) and the kidneys containing the engrafted TPs were harvested. Tissues were fixed in 4% paraformaldehyde (PFA) at 4 °C for 24 h, followed by dehydration and embedding in paraffin. Serial sections (5 μm thick) were prepared for analysis.

For immunofluorescence, the sections were deparaffinized, rehydrated, and subjected to heat-induced antigen retrieval in citrate buffer (pH 6.0). After blocking with 5% BSA and 0.3% Triton X-100 for 1 h, the slides were incubated with primary antibodies against thyroid markers (TG) at 4 °C overnight. Following incubation with Alexa Fluor-conjugated secondary antibodies (1:350) and DAPI counterstaining, images were captured using a Zeiss LSM980 confocal microscope, and the images were processed using Photoshop or Zeiss Zen.

### Statistical analyses

This study was designed as a protocol-optimization study in which sample sizes were determined on the basis of prior pilot data and practical feasibility rather than formal *a priori* power calculation. The control group (n = 85 grafts) represents the cumulative tumor incidence across three sequentially developed protocols (Protocols 1–3), whereas the experimental group (n = 10 grafts) reflects a single optimized protocol (Protocol 4); the difference in group size reflects this cumulative-versus-single-protocol study design rather than unequal allocation. The primary endpoint—tumor incidence rate—was compared between groups using a binary outcome with a large expected effect size (82.35% vs. 10%), for which the observed difference remained highly significant (Fisher’s exact test, P < 0.0001) despite the limited cohort size. All continuous data, including RT-qPCR results, are descriptive (presented as mean ± SD, n=3) and were not subjected to formal statistical testing. Data visualization and analyses were conducted using Prism software (v8, GraphPad). Animals were randomly assigned to experimental groups, and data collection and analyses were performed by investigators blinded to the treatment conditions. We acknowledge that for future studies aimed at detecting more subtle functional differences, formal sample-size calculations will be required.

## Data Availability

The raw data supporting the conclusions of this article will be made available by the authors, without undue reservation.
