# Supplementary material for: Stage-specific signaling modulation generates low-tumorigenicity thyroid progenitors from human pluripotent stem cells
Source: Front Endocrinol (Lausanne). 2026 Aug 31;17:1885613. doi: 10.3389/fendo.2026.1885613 (PMC13572144; doi:10.3389/fendo.2026.1885613)
Supplement: Supplementary file 1 [file SupplementaryFile1.docx]

Supplementary Material

**Stage-Specific Signaling Modulation Generates Low-Tumorigenicity Thyroid Progenitors from Human Pluripotent Stem Cells**

Lili Dong^1†^, Manzhen Peng^1†^, Chuanyu Yao^1^, Xie Cheng^1^, Haisong Liu^1, 2*^

1 School of Biomedical Sciences, Hunan University, Changsha, Hunan, China.

2 Xiangtan Central Hospital, Affiliated to Hunan University, Xiangtan, Hunan, China.

*** Correspondence:** Haisong Liu

liuhaisong@hnu.edu.cn.

^†^ Lili Dong, Manzhen Peng contributed equally to this work.

**This file includes:**

Supplementary Figure Legends

Supplementary Tables

**Supplementary Figure Legends**

**Supplementary Figure 1. Gene characterization of H1 differentiation to AFE and small-molecule screening matrix for thyroid follicular maturation.**

(A) Relative mRNA expression levels of the pluripotency markers *NANOG* and *OCT4* in undifferentiated *PAX8*-mCherry cells. Data are presented as mean ± SD (n = 3).
(B) Relative mRNA expression levels of the anterior foregut endoderm markers (*PAX9*, *SOX2*, and *HHEX*) in H1 and AFE cells. Data are presented as mean ± SD (n = 3).
(C) Matrix of the 12 small-molecule combinations systematically evaluated during the extended *in vitro* maturation phase (post-Day 15) to consolidate thyroid follicular identity and 3D architecture.
(D) Immunofluorescence of grafts at Day 90 post-transplantation from Protocol 1. Grafts remain disorganized, lacking organized TG⁺ follicular structures. Dashed lines delineate the boundary between the host kidney and the graft. Scale bars, 100 μm.

**Supplementary Figure 2.** **Hedgehog pathway activation via SAG exerts minimal effects on thyroid progenitor specification.**

(A) RT-qPCR analysis of *NKX2-1*, *PAX8*, and *FOXE1* transcript levels in Day-15 thyroid progenitors differentiated in the absence or presence of SAG supplementation. Relative mRNA levels are shown normalized to *GAPDH*. Data represent mean ± SD (n = 3).

(B) Representative FACS plots quantifying *PAX8*-mCherry⁺ populations at Day 15 in the absence (left, 12.0%) or presence (right, 18.9%) of SAG supplementation during Stage 3. Gates were set based on undifferentiated H1 ESC controls. Percentages indicate the proportion of positive cells from a single experiment (n = 1).

**Supplementary Figure 3. Evaluation of small-molecule combinations during S3a/S3b and S2b stages, *in vivo* tumorigenicity assessment, and flow cytometry gating strategy.**

(A) Matrix detailing the 16 small-molecule combinations evaluated across the S3a and S3b windows.

(B) Representative immunofluorescence images of kidney grafts at Day 45 post-transplantation of cells generated under alternative high-performing S3a/S3b conditions, demonstrating the emergence of teratomatous structures, underscoring that tumorigenicity is a common feature across multiple optimized induction regimens. Scale bars, 100 μm.

(C) Matrix of the 16 small-molecule combinations systematically evaluated during the S2b window of anterior foregut endoderm specification.

(D) Gating strategy and negative control for intracellular flow cytometry. Left: Debris exclusion (FSC-A vs. SSC-A). Middle: Single-cell gating based on DNA content (DAPI). Right: Negative control demonstrating minimal non-specific binding (<1.1%), confirming the specificity of the overnight intracellular staining.

**Supplementary Tables**

**Supplementary Table 1: Composition and Preparation of MS12^(1)^ (800 mL batch)**

| **Component** | **Vo****lume** |
| --- | --- |
| MCDB131 | 744.8 mL |
| 45% glucose | 3.2 mL |
| 20% Bovine Serum Albumin (fatty acid-free) | 20 mL |
| 7.5% sodium bicarbonate | 16 mL |
| GlutaMax | 8 mL |
| Penicillin-Streptomycin | 8 mL |
| Total | 800 mL |

**Supplementary Table** **2: Composition and Preparation of cSFDM^(2, 3)^ (500 mL batch)**

| **Component** | **Final Concentration** | **Volume** |
| --- | --- | --- |
| IMDM | 75% | 365.1 mL |
| Ham’s F12 | 25% | 121.7 mL |
| N-2 Supplement | 0.5× | 2.5 mL |
| B-27 Supplement (with RA) | 0.5× | 2.5 mL |
| Bovine Serum Albumin (fatty acid-free) | 0.05% | 2.5 mL |
| L-ascorbic acid (0.05 mg/mL) | 0.05 μg/mL | 500 μL |
| 0.45 mM Monothioglycerol | 0.45 mM | 250 μL |
| PS | 1× | 5 mL |
| Total |  | 500 mL |

Abbreviations: PS, penicillin-streptomycin; IMDM, Iscove's Modified Dulbecco's Medium; RA, retinoic acid.

**Supplementary Table 3: List of primer sequences used for RT-qPCR analysis**

| **Gene Name** | **Primer Forward** | **Primer Reverse** | **Species** |
| --- | --- | --- | --- |
| *GAPDH* | GACTCATGACCACAGTCCATGC | AGAGGCAGGGATGATGTTCTG | Human |
| *NKX2-1* | CAGGACACCATGAGGAACAGC | CCCATGAAGCGGGAGATG | Human |
| *PAX8* | CGAGCGACTCCCCGGCGAT | GAGGTCTGCCATTCACAA | Human |
| *FOXE1* | TTCTACCGCGACAACCCCAA | TCGCGCGGGATCTTGAGGAA | Human |
| *TG* | GGCTAATGCTACATGTCCTG | GCTTCTGTTGGAGATGCTGG | Human |
| *TPO* | GTCTGTCACGCTGGTTATGG | CAATCACTCCGCTTGTTGGC | Human |
| *TSHR* | GAACTGATAGCAAGAAACACCTGG | GTATCCTGGAACTTGGACTTTT | Human |
| *NIS* | TGTCTGCCAGCTTCATGTCG | GGTGTACAGCATCGTGGCTA | Human |
| *IRX1* | CCCGGGGGCACTCAATG | TCTGGCCTGGGGACGAG | Human |
| *TBX1* | CCCACGCAAAGATAGCGAGA | CTGCGTGATCCGATGGTTCT | Human |
| *SOX17* | GTGGACCGCACGGAATTTG | GGAGATTCACACCGGAGTCA | Human |
| *FOXA2* | CTGAGCGAGATCTACCAGTGGA | CAGTCGTTGAAGGAGAGCGAGT | Human |
| *SOX2* | GCTACAGCATGATGCAGGACCA | TCTGCGAGCTGGTCATGGAGTT | Human |
| *PAX9* | AGCAGGAAGCCAAGTACGGTCA | CAGAAGGAGCAGCACTGTAGGT | Human |
| *HHEX* | AGGAAGCGTCTGGCCAAGAT | ATTGCTTTGAGGGTTCTCCTGTT | Human |
| *OCT4* | CCGAAAGAGAAAGCGAACCAG | ATGTGGCTGATCTGCTGCAGT | Human |
| *NANOG* | CACCCAGCTGTGTGTACTC | CTTCTGCGTCACACCATT | Human |
| *HEY1* | TGTCTGAGCTGAGAAGGCTGGT | TTCAGGTGATCCACGGTCATCTG | Human |
| *PAX2* | GACTATGTTCGCCTGGGAGATTC | AAGGCTGCTGAACTTTGGTCCG | Human |
| *WT1* | CGAGAGCGATAACCACACAACG | GTCTCAGATGCCGACCGTACAA | Human |
| *TUBB3* | TCAGCGTCTACTACAACGAGGC | GCCTGAAGAGATGTCCAAAGGC | Human |
| *SOX1* | GAGTGGAAGGTCATGTCCGAGG | CCTTCTTGAGCAGCGTCTTGGT | Human |

| **Primary antibodies** | | | | | |
| --- | --- | --- | --- | --- | --- |
| **Protein** | **Provider** | **Catalog number** | **Host Species** | **Dilution**  **Flow Cyt.** | **Dilution**  **IF** |
| Nkx2.1 | Abcam | AB76013 | Rabbit | 1:200 | 1:200 |
| Nkx2.1 | ThermoFisher | MA5-13961 | Mouse | 1:200 | 1:200 |
| Pax8 | Proteintech | 21383-1-AP | Rabbit | 1:1500 | 1:1000 |
| Tg | Abcam | AB187378 | Mouse | 1:500 | 1:200 |
| mCherry | Abcam | ab05402 | Chicken | 1:300 | 1:300 |
| **Secondary antibodies** | | | | | |
| Donkey anti-Mouse Alexa Fluor 488 | Jackson | 715-545-150 |  | 1:350 | 1:350 |
| Donkey anti-Rabbit Alexa Fluor 488 | Jackson | 711-545-152 |  | 1:350 | 1:350 |
| Donkey anti-Mouse Alexa Fluor 555 | Beyotime | A0460 |  | 1:350 | 1:350 |
| Donkey anti-Rabbit Alexa Fluor 555 | Jackson | 711-565-152 |  | 1:350 | 1:350 |
| Donkey anti-Chicken Alexa Fluor 555 | Jackson | 703-565-155 |  | 1:350 | 1:350 |
| Donkey anti-Mouse Alexa Fluor 647 | Jackson | 715-605-151 |  | 1:350 | 1:350 |
| Donkey anti-Rabbit Alexa Fluor 647 | Jackson | 711-605-152 |  | 1:350 | 1:350 |

**Supplementary Table 4: List of primary and secondary antibodies used in the experiment**

**References**

1.	Liu H, Li R, Liao HK, Min Z, Wang C, Yu Y, et al. Chemical combinations potentiate human pluripotent stem cell-derived 3D pancreatic progenitor clusters toward functional beta cells. Nat Commun. 2021;12(1):3330.doi: 10.1038/s41467-021-23525-x

2.	Undeutsch HJ, Posabella A, Alber AB, Bawa PS, Villacorta-Martin C, Wang F, et al. Derivation of transplantable human thyroid follicular epithelial cells from induced pluripotent stem cells. Stem Cell Reports. 2024;19(12):1690-705.doi: 10.1016/j.stemcr.2024.10.004

3.	Kurmann AA, Serra M, Hawkins F, Rankin SA, Mori M, Astapova I, et al. Regeneration of Thyroid Function by Transplantation of Differentiated Pluripotent Stem Cells. Cell Stem Cell. 2015;17(5):527-42.doi: 10.1016/j.stem.2015.09.004
